# One Health: enabler of effective prevention, control and elimination of emerging and re-emerging infectious diseases

**DOI:** 10.1186/s40249-025-01337-1

**Published:** 2025-07-31

**Authors:** Tianyun Li, Xiao-Nong Zhou, Marcel Tanner

**Affiliations:** 1 School of Global Health, Chinese Center for Tropical Diseases Research, Shanghai Jiao Tong University School of Medicine, Shanghai, 200025 China; 2 School of Public Health, Shanghai Jiao Tong University School of Medicine, Shanghai, 200025 China; 3 National Key Laboratory of Intelligent Tracking and Forecasting for Infectious Diseases, National Institute of Parasitic Diseases, Chinese Center for Disease Control and Prevention (Chinese Center for Tropical Diseases Research), NHC Key Laboratory for Parasitology and Vector Biology, WHO Collaborating Centre for Tropical Diseases, National Centre for International Research On Tropical Diseases, Shanghai, 200025 China; 4 Hainan Tropical Diseases Research Center (Hainan Sub-Center, Chinese Center for Tropical Diseases Research), Haikou, 571100 China; 5 Swiss Tropical and Public Health Institute, 4123 Allschwil, Switzerland; 6 University of Basel, 4003 Basel, Switzerland

**Keywords:** Emerging infectious diseases, Re-emerging infectious diseases, One Health, Public health, Ecosystems health, Systems thinking

## Abstract

Emerging and re-emerging infectious diseases in different soci-ecological settings create unprecedented challenges for global public health and socio-economic development. The One Health concept is based on a systemic, transdisciplinary approach and hence captures the interactions between humans and animals, in a given socio-ecological setting. It could comprehensively address the human-animal-environment interface, the core of zoonotic diseases. Consequently, One Health approach is effective in controlling and eliminating the promoting factors of emerging infectious diseases (EIDs). We explored key principles for the prevention, control and elimination of EIDs through reviewing the transition of public health and global health strategies towards One Health and summarizing some successful experiences in effectively controlling EIDs. Recognizing heterogeneities and strengthening “surveillance-response systems” are the two key principles. It is recommended to promote health equity and conduct cost-effectiveness analysis to address the challenges of heterogeneity. Cross-sectoral collaboration and transdisciplinarity should be strengthened to facilitate the utilizing of systems thinking.

**Graphical abstract:**

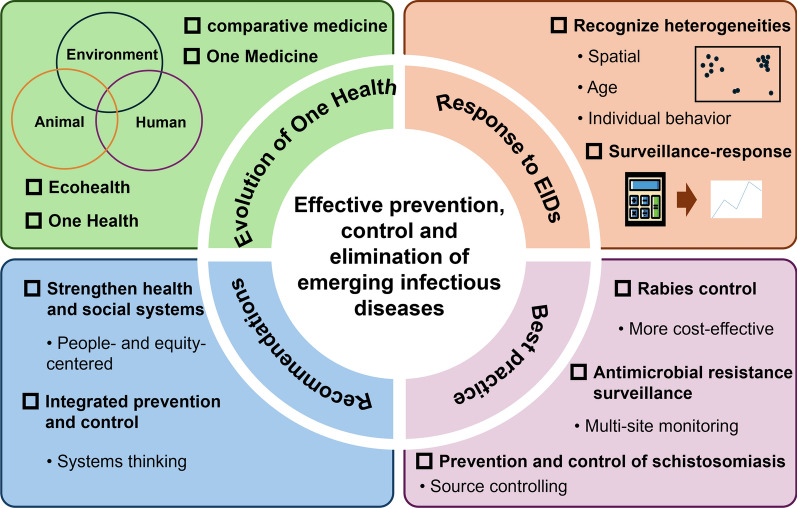

## Background

### Emerging infectious diseases

Emerging infectious diseases (EIDs) are diseases that have seen a rise in human cases over the last 20 years or show potential for a future surge [1], including diseases caused by newly evolved pathogens and those newly infecting humans. Re-emerging infectious diseases refer to diseases caused by known pathogens in humans with rising incidence [2]. Risk factors of them include frequency interactions between humans and animals, increased population density and mobility, poverty and social inequality, ecosystem changes as a consequence of climate change and loss of biodiversity [3]. In addition, over 60% of infectious diseases are zoonotic [4]. Clearly, the human-animal-environment interface invites EIDs and re-emerging infectious diseases.

EIDs pose a great challenge to global public health. To date, the transmission of EIDs is displaying new patterns. Taking Ebola as an example, previous outbreaks in Africa occurred in remote, rather isolated settings and were characterized by low population densities and mobility, and existing peripheral health services with no real surveillance systems. However, recent major outbreaks occurred in less isolated settings with high population densities, high population mobility and weak health systems [5]. Moreover, EIDs are more likely to place a serious burden due to the lack of surveillance and response systems, especially in low- and middle-income countries [6]. For example, coronavirus (SARS-CoV-2) and the related disease COVID-19, an EID originated with bats and spreading through the contact between humans and wildlife [7], has caused 776 million cases and more than 7 million deaths globally so far [8]. An urgent priority is identifying applicable public health strategies for responding to EIDs.

### The evolution of One Health

**Fig. 1:**
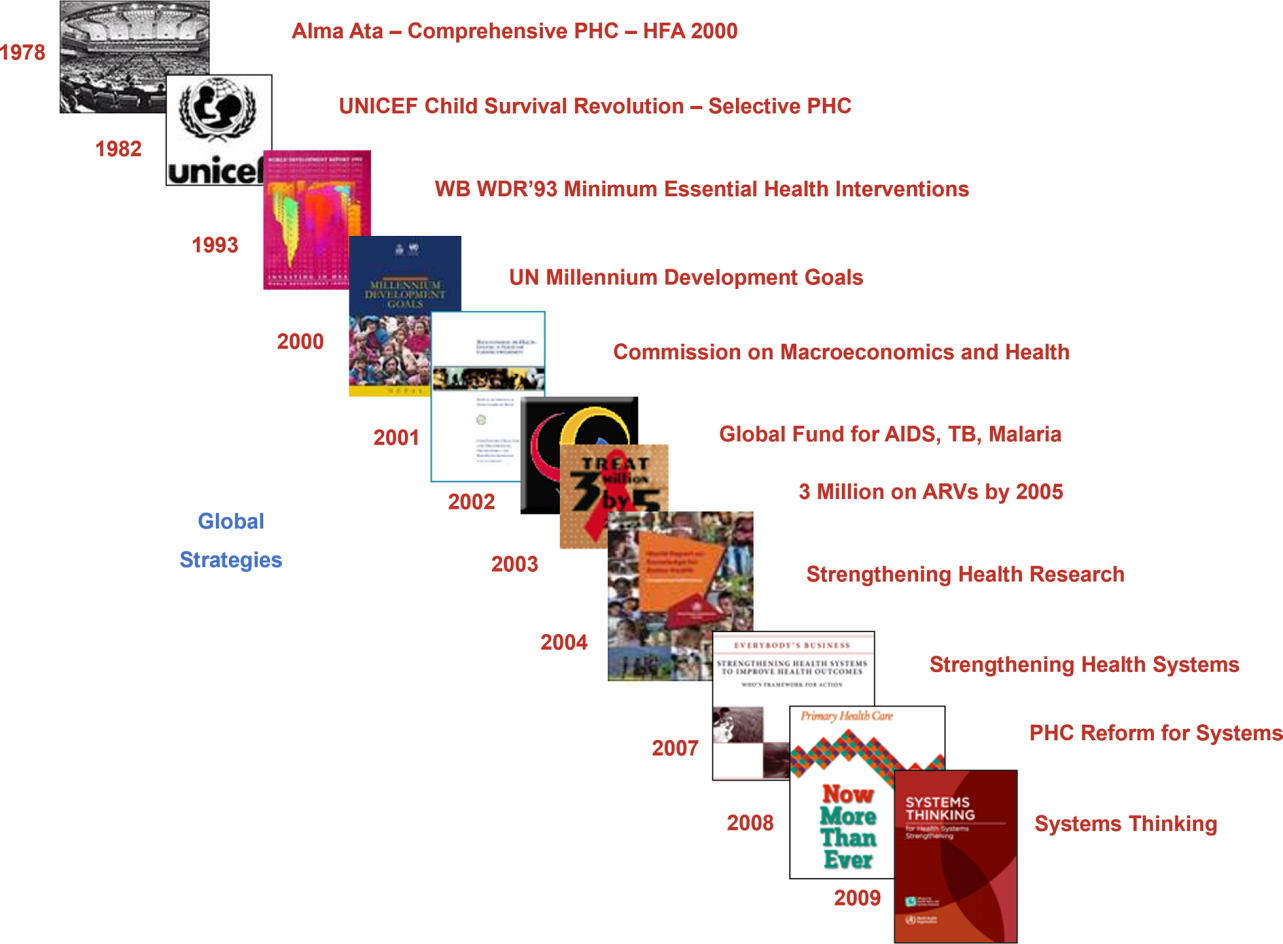
The development of global strategies as response to the global health challenges over the past 50 years [11]. The pictures show the cover pages of the respective international reports; Abbreviations: *AIDS* Acquired immunodeficiency syndrome, *ARVs* antiretrovirals, *HFA* Health for all by the year 2000, *HPSR* Health Policy and Systems Research, *PHC* Primary Health Care, *TB* Tuberculosis, *UN* United Nations, *UNICEF* United Nations Children’s Fund, *WB* World Bank, *WDR* World development report, *WHO* World Health Organization

Throughout the history of public health, global strategies have reflected a gradual shift toward the One Health approach (Fig. 1). Typically, primary health care (PHC) was put forward in 1978, emphasizing equitable access and multisectoral action [9]. The World Development Report of 1993 shifted focus from maximizing good health to reducing burden. In 2000, Millennium Development Goal 7 proposed to reduce the burden of diseases through improving sanitation facilities, which linked human health to environment. The World Health Report 2008 further advanced the principles of universal access, equity and social justice to response to health challenges [10]. In 2009, systems thinking was considered important to design and evaluate health interventions. In 2015, the Sustainable Development Goals (SDGs) re-emphasized the impact of economic, social and environmental factors on human health.

Initially, human and animal diseases were treated by so-called “healers” and medicine had no clear species boundaries [12] until the emergence of veterinary medicine in 1762. The “comparative medicine” emerged in the twentieth century using animals as research models, indicating the unification of human medicine and veterinary medicine [13]. “Ecohealth”, a concept developed in the 1970s, emphasized the crucial interactions between ecosystems and human health [14]. Then “One Medicine” was put forward by Calvin W. Schwabe based on work with Dinka pastoralists in Sudan [15]. “One World, One Health” was first put forward in 2004 at the meeting organized by the US-based Wildlife Conservation Society. Nowadays, One Health serves as an integrated approach to response to the complex global public health issues through balancing the health of people, animals and the environment [16], which is considered a feasible approach for dealing with EIDs.

Despite the adoption of the One Health concept in policy-making, practical implementation through One Health approach remains challenging. Outlining core One Health principles for implementation could enhance policy enforcement. Therefore, this article aimed to explore public health strategies for the prevention, control, and elimination of EIDs through identifying the key principles of taking actions based on the One Health concept and learning from successful practices.

## Prevention, control and elimination of emerging and re-emerging infectious diseases within One Health

There are two key principles that guide global public health actions; particularly the control and possible subsequent progressive elimination.

### Recognizing heterogeneities

Recognizing spatial, age, and individual behavioral heterogeneity of EIDs is essential for adopting appropriate public health strategies. Regional heterogeneity is influenced by the spatial and social structure of the population [17]. For example, enteric infections have consistently had the highest disease burden in low-income countries, largely as a result of poor sanitation [18]. In addition, different age groups have different levels of infection and complications from the same pathogens; for example, global dengue cases are concentrated in adolescent and elderly populations [19, 20]. Moreover, dietary habits, personal hygiene, social interaction rates, and culturally influenced customs all affect the spread of infectious diseases [21]. Diseases transmitted through droplets and aerosols tended to show higher infection rates in areas with higher social interaction rates [22]. Rapid human movement demonstrated significant correlation with increased dengue transmission in Bangladesh [23].

The prevention and control of EIDs should focus on the heterogeneity of disease transmission, which leads to immediate and effective responses in specific socio-ecological contexts [22] and enhances the resilience of the public health system. There are many well-established quantitative methods available for analyzing the outbreak, transmission, and control of infectious diseases, such as risk factor analysis, risk modeling, and dynamic modeling [24]. Parameters related to heterogeneity should be incorporated into the model to ensure comprehensive and targeted strategies, which is considered a great challenge.

### Surveillance-response strategy

The “surveillance-response systems” based on One Health concept should integrate human, animal, and environmental health data through near real-time collection, processing, and feedback to enhance the accuracy and sustainability of public health decision-making. The “First Forum on Surveillance-Response System Leading to Tropical Diseases Elimination” held in 2012 in Shanghai, China indicated that such systems require validated, evidence-based minimum essential dataset (MED) incorporating spatiotemporal dimensions [25], supported by highly sensitive diagnostic tools to detect low-prevalence and emerging pathogens [26]. Applying MED to develop context-specific transmission models and conduct intervention assessments—accounting for environmental heterogeneity—could significantly improve emergency response efficiency while optimizing the allocation of limited public health resources [25, 27]. This system should be made possible at subnational levels, such as regional, provincial and district levels, particularly in decentralized health systems. In addition, both surveillance systems and responsive interventions should be (i) integrated and (ii) tailored to transmission settings, i.e. the socio-ecological settings. However, the COVID-19 pandemic revealed critical gaps in global preparedness, irrespective of economic level. Clearly, a lot of data are collected, which are considered best available data, but there is nearly no surveillance systems based on real-time MED in space and time to detect changes/unusual situations.

## Best practice: three telling examples

Without entering into details, we summarized three examples that convincingly demonstrated the essence and usefulness of surveillance-response strategy within a One Health approach in diseases control and elimination.

### Antimicrobial resistance surveillance

Antimicrobial resistance (AMR) represents a growing public health crisis, with interconnections and interactions between humans, animals and the environment driving the emergence and spread of antibiotics and drug-resistant bacteria [28]. As early as 2002, the Public Health Agency of Canada launched the initiative of Canadian Integrated Program for Antimicrobial Resistance Surveillance (CIPARS), which was an outstanding example of integrated surveillance systems. This comprehensive program was designed to monitor AMR in bacteria isolated from humans and livestock along the “farm-to-fork” food chain. Farms, abattoirs, retail foods, and clinical isolates from animals and humans were the main targets for monitoring [29, 30]. It is of high time that countries get inspired by the leading example of CIPARS and implement One Health strategies against AMR. An effective “surveillance-response system” should be developed by shifting from collecting best available data to MED, enabling comprehensive analysis through integrated multi-source data. Simultaneously, strengthen the cooperation and communication among environment, agriculture, food, and public health sectors to enhance joint response.

### Rabies control

Rabies is a typical zoonotic disease which is transmitted to humans mainly through the bite from dogs with rabies. Since 2000, the Swiss Tropical and Public Health Institute has worked closely with two Chadian partner institutions in N’Djamena, including the Institute de Recherche en Elevage pour le Développement and the Centre de Support en Santé International, to demonstrate the feasibility of dog vaccination [31]. Zinsstag et al. compared human rabies incidence and cost-effectiveness between mass dog vaccination and post-exposure prophylaxis (PEP). A single parenteral dog rabies-mass vaccination campaign (≥ 70% coverage) could interrupt transmission of rabies from dogs to humans for at least 6 years. In particular, combining parenteral dog-vaccination campaigns with human PEP proved more cost-effective than human PEP alone [32]. Therefore, the transdisciplinary and cross-sectoral collaboration between public health and animal health should be strengthened to shift the focus of disease control from treating human cases to interrupting cross-species transmission. Furthermore, cost-effectiveness analysis should be conducted to determine public health strategies and resource allocation.

### Prevention and control of schistosomiasis

Schistosomiasis, a neglected tropical disease caused by *Schistosoma* spp., spreads through contact with infected water. China once suffered from the heaviest burden of schistosomiasis with 11.6 million infected people [33]. Control efforts occurred in three phases: (1) Mid-1950s–1980s: Snail elimination via environmental/chemical interventions; (2) 1980s–2003: Large-scale drug administration for human/livestock and continued snail control; (3) 2004–present: Expanded multisectoral (agriculture, water conservation, natural resources, and forestry) collaboration, and enhanced snail/livestock/water management. By 2015, endemic areas achieved < 1% of human infection rates, and by 2023 achieved 0 in human infection rates in all endemic counties [37]. It is evident that enhance cross-sectoral coordination at human-animal-environment interface in lens of One Health could effectively interrupt the transmission of pathogens to humans. This requires establishing standardized implementation protocols to guide coordinated actions, with clearly defined responsibility boundaries across sectors to ensure effective execution.

## Recommendations from One Health perspective

### Strengthen resilience of surveillance system

**Fig. 2:**
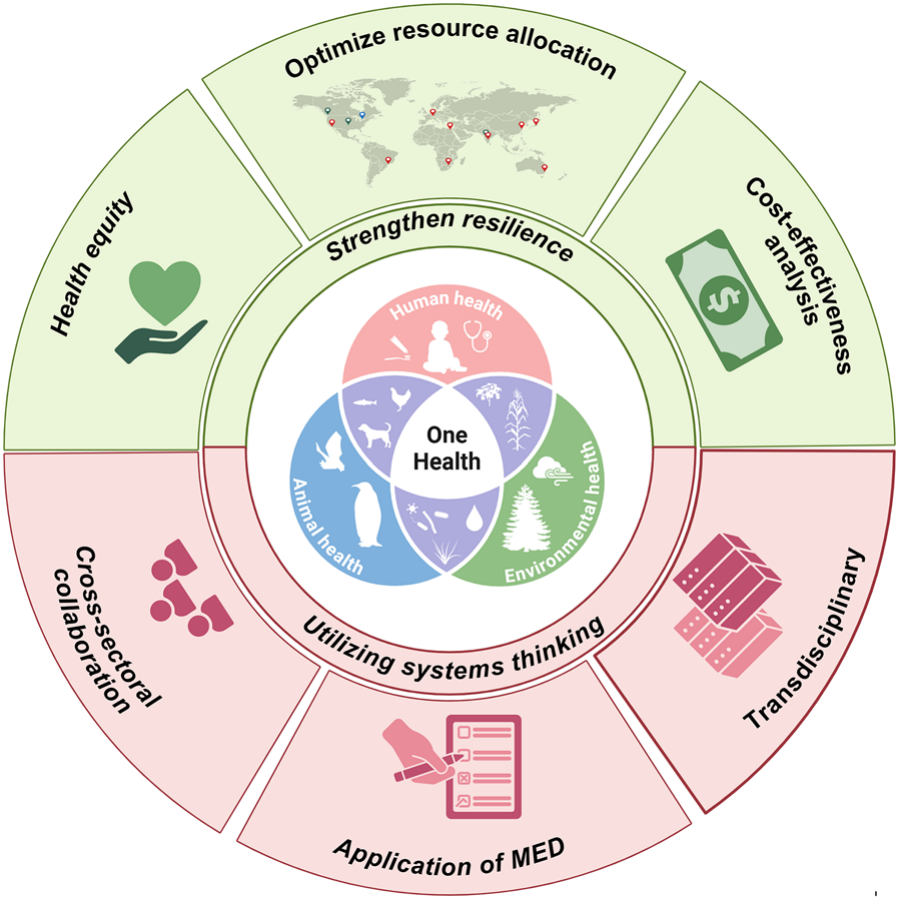
Recommendations for prevention, control and elimination of emerging and re-emerging infectious diseases from One Health perspective. Abbreviation: *MED* minimum essential dataset

Enhancing health system resilience by promoting health equity is critical for dealing with the challenges posed by heterogeneity of EIDs (Fig. 2). Health equity means that all people, rich and poor, have access to quality and affordable health care [34]. Strengthening cross-regional cooperation through the One Health approach could facilitate the transfer of advanced disease prevention and control experiences to countries with higher disease burden.

Recognize the spatially heterogeneous characteristics of disease through One Health approach to guide the precise allocation of resources. Focus should always be on improving health systems in economically disadvantaged areas; within countries and in the different regions of the world. National and international organizations should prioritize economic and technical assistance to these regions. A previous study predicted the future risk of disease in geographical locations in Hong Kong by analyzing the spatial and temporal transmission patterns of COVID-19 as a basis for prioritizing resource allocation [35], which is considered a paradigm.

Conduct cost-effectiveness analysis to optimize health strategies. Reducing investment while ensuring the quality of interventions is the key. The control and prevention of Malaria provided a good practice in this regard. A transmission dynamics model was developed to analyze the effectiveness and cost-effectiveness of three control strategies, and the most cost-effective strategy was found to be a combination of insecticide spraying and treatment of infected individuals [36].

### Utilizing systems thinking for integrated prevention and control

Systems thinking based on the One Health concept emphasizes the interdependence of human, animal and environmental health. Disease prevention and control guided by systems thinking is not at the expense of animal and environmental health, which is effective and sustainable. Cross-sectoral collaboration and transdisciplinary are critical for applying systems thinking to response to EIDs (Fig. 2).

Strengthen multisectoral cooperation and communication through the establishment of collaborative mechanisms. Surveillance systems should cover human, animal and environment, especially the health of the interfaces across them, which contain high risk for pathogen spills [37]. Collaboration across multiple sectors is essential for data-sharing and integration of resources. Surveillance for animal and environment in lens of One Health may be able to detect possible disease risks at an early stage. For example, the COVID-19 pandemic revealed the great potential of environmental monitoring of wastewater, and many countries have now adopted wastewater pathogen surveillance for many public health-related diseases [38, 39]. The establishment of a MED should take into account human, animal, and environmental-related factors in order to make a comprehensive assessment of EIDs.

It is recommended to use transdisciplinary science to address challenges posed by EIDs. Transdisciplinary science, as distinct from multidisciplinary and interdisciplinary approaches, emphasizes the integration of knowledge from different disciplines to create new paradigms and methods [40]. In order to fully understand and respond to EIDs, human medicine, veterinary medicine, public health, biology, environmental sciences, plant sciences, social sciences, etc. should be taken into account. These disciplines have their own complete knowledge systems, and the perspectives on the same issue could be heterogeneous under different disciplinary thinking. Transdisciplinary primarily requires the capacity of creative listening among all partners, even more than just expertise and experience.

## Conclusions

As climate change intensified, extreme weather events such as global warming, droughts, and acid rain affects animal habitats and activity ranges, increasing human-animal interactions and heightening the risk of pathogens spilling over from the environment and animals to humans. The One Health approach is part of a solution to mitigate these effects through recognizing heterogeneities and strengthening “surveillance-response systems”. It is recommended to promote health equity and conduct cost-effectiveness analysis to address the challenges of heterogeneity. Cross-sectoral collaboration and transdisciplinarity should be strengthened to facilitate the utilizing of systems thinking.

## Data Availability

Not applicable.

## Abbreviations

AMR: Antimicrobial resistance
CIPARS: Canadian integrated program for antimicrobial resistance surveillance
EIDs: Emerging infectious diseases
MED: Minimum essential dataset
PEP: Post-exposure prophylaxis
PHC: Primary health care
SDGs: Sustainable Development Goals
